# Characterization of a Human Antibody Fragment Fab and Its Calcium Phosphate Nanoparticles that Inhibit Rabies Virus Infection with Vaccine

**DOI:** 10.1371/journal.pone.0019848

**Published:** 2011-05-09

**Authors:** Xinjian Liu, Hong Lin, Qi Tang, Chen Li, Songtao Yang, Zhongcan Wang, Changjun Wang, Qing He, Brian Cao, Zhenqing Feng, Xiaohong Guan, Jin Zhu

**Affiliations:** 1 Key Laboratory of Antibody Technique of Ministry of Health, Nanjing Medical University, Nanjing, China; 2 Huadong Medical Institute of Biotechniques, Nanjing, China; 3 Van Andel Institute, Antibody Technology Lab, Grand Rapids, Michigan, United States of America; 4 Veterinary Institute of the Academy of Military Medical Sciences, Changchun, China; 5 Department of Pathology, Nanjing Medical University, Nanjing, China; University of California, Merced, United States of America

## Abstract

Recombinant antibody phage display technology has been used to mimic many aspects of the processes that govern the generation and selection of high-affinity natural human antibodies in the human immune system, especially for infectious disease prophylaxis. An anti-rabies virus immunized phage-display Fab library was constructed from peripheral blood lymphocytes from vaccinated volunteers. The immunized antibody library, with a diversity of 6.7×10^8^, was used to select and produce antibodies that bound to rabies virus glycoprotein. After five rounds of immobilized fixed rabies virion panning, four unique DNA sequences were found in the higher binding clones, and only one, Fab094, showed neutralization activity. Fab094 components were analyzed by ELISA, immunoprecipitation and immunofluorescent staining. ELISA and immunofluorescence showed that Fab094 bound specifically to rabies virions. Immunoprecipitation and mass spectrometry showed that Fab094 reacted with rabies virus glycoprotein. To improve the penetration power of Fab094 antibodies, we developed Fab094 calcium phosphate nanoparticles (Fab094-CPNPs) and tested their efficacy. The rapid fluorescent focus inhibition test indicated that the neutralizing antibody titers of Fab094 and Fab094-CPNPs were reached at 200.17 IU/Kg and 246.12 IU/Kg, respectively. These findings were confirmed in vivo in a Kunming mouse challenge model. Our results demonstrate that human Fab094 and Fab094-CPNPs are efficacious candidate drugs to replace rabies immunoglobulin in post-exposure prophylaxis (PEP).

## Introduction

Rabies is a zoonotic viral disease that infects wild as well as domestic animals [1]. It is estimated that at least 500,000 people receive post-exposure vaccination and that 55,000 people die from rabies each year [2], especially in Africa and Asia where rabies is endemic, and where successful canine rabies vaccination or control programs have not been implemented [3]. According to the categorization of exposure defined by the World Health Organization (WHO), the most severe cases (category III) require wound cleaning, rabies vaccination, and direct wound infiltration with rabies immunoglobulin (RIG). Both purified equine rabies immunoglobulin (ERIG) and human immunoglobulin (HRIG) are used in rabies endemic areas [3], [4]. ERIG that is manufactured presently is highly purified and the occurrence of adverse events has been reduced significantly, but serious reactions, including anaphylaxis and serum sickness caused by heteroantigens, can occur in spite of a negative skin test [5]. HRIG is purified from carefully selected donors, and processing eliminates viral contaminants, but it still can increase susceptibility to various infections, including HIV and hepatitis viruses.

Alternatives to HRIG and ERIG should be considered, including human monoclonal antibodies, human recombinant antibodies [6], and antibodies from other animals, such as sheep [7]. Ray et al. have described two rabies-virus-neutralizing scFv–Fc fusion proteins isolated from a human synthetic scFv phage display library [8]. Ando et al. have reported two Fab preparations, EP5G3 and GD2D12, that were isolated from a phage display library, which have neutralizing activity against rabies virus strain CVS when assayed by rapid fluorescent focus inhibition test (RFFIT) [1]. Houimel et al. also have reported three Fabs isolated from a recombinant immune antibody library [2]. However, the neutralizing activity of these Fab antibodies has not been confirmed *in vivo*.

In recent years, new strategies for cancer treatment based on drug-loaded nanoparticulate formulations have emerged [9]. Nanoparticles are promising drug carriers that show high drug-loading efficiency, minor drug leakage, and good storage stability, and they can circumvent multidrug resistance of cancer cells [10]. Above all, nanoparticles have an enhanced permeability and retention (EPR) effect [11]. Moreover, their body biodistribution and permeability in tissues can be controlled by size and surface properties [12].

The current study describes the isolation of human Fabs with rabies-virus-neutralizing activity from a human immunized phage display library using peripheral blood lymphocytes. In addition, we developed Fab094-calcium phosphate nanoparticles (CPNPs) and tested their efficacy *in vitro* neutralization assay and animal model *in vivo*.

## Materials and Methods

### Rabies strains and cells

Rabies virus strain CTN (which has 83.2–96.8% nucleic acid and 90.0–97.4% amino acid sequence homology to street strains [13]), was provided by Wuhan Institute of Virology, Chinese Academy of Sciences. Rabies virus strain CVS-11 and BHK-21 cells were from the Veterinary Institute of the Academy of Military Medical Sciences, China. BHK-21 cells were cultured in DMEM (Gibco, USA) supplemented with 10% fetal bovine serum (FBS, Gibco, USA). Cell lines were maintained at 37°C under 5% CO_2_.

### Animals

Kunming mice (10–12 g) were obtained from the Experimental Animal Center of the Academy of Military Medical Sciences of China. All animal breeding and experiments were approved by the Veterinary Institute of the Academy of Military Medical Sciences animal Ethics Committee [Project Numbers SYXK (ARMY) 2009- 045].

### Preparation of cDNA for library construction

Lymphocytes were collected from 45 healthy donors who were immunized with rabies vaccine (Flury LEP; Chiron Behring Vaccines Pvt. Ltd). The volunteers got their signed, informed consent to participate, and the Ethics Committee of Nanjing Medical University approved the study.

Up to 10 ml anticoagulant blood was diluted with 10 ml PBS. Human peripheral blood mononuclear cells were isolated on a Ficoll-Pacque gradient and total RNA was prepared by using an RNA Purification kit (QIAGEN, Valencia, CA, USA). First-strand cDNA was synthesized from total RNA by using a First-strand cDNA Synthesis kit (Invitrogen, USA) with Oligo-dT18.

### Construction of Fab library

For the amplification of Fab gene segments, a unique three-step PCR was used [14]. The V regions of heavy and light chains, C_H_1 (with IgG1 isotype) and C_L_ (including κ and λ) were amplified first. In a second step, the amplified V_H_, V_L_, C_H_1 and C_L_ were joined together in an overlap PCR to amplify the Fd and light chains. The Fd and L chains were mixed in equal ratios to generate full-length Fab fragments. The light- and heavy-chain Fds were spliced by PCR overlap extension with primers appended with *Sfi*I restriction sites, as described above. The resultant Fab was digested with *Sfi*I (Roche Molecular Biochemicals, Mannheim, Germany), purified on agarose gel, and ligated into the phagemid pComb3XSS (provided by the Barbas III Laboratory) that had been cut with the same restriction enzyme.

After ligation, DNA (2 µg) was ethanol-precipitated, resuspended in 15 µl of water, and electro-transformed into 300 µl *Escherichia coli* XL1-Blue (Stratagene, La Jolla, CA, USA). After transformation, 5 ml SOC medium was added at room temperature, and the cultures were shaken at 300 rpm for 1 h at 37°C. After addition of 10 ml pre-warmed (37°C) SB medium that contained 20 µg/ml ampicillin and 10 µg/ml tetracycline, the cultures were shaken at 300 rpm for an additional l h. These cultures were added to 180 ml pre-warmed SB medium that contained 50 µg/ml ampicillin and 10 µg/ml tetracycline, after which, 2 ml helper phage VCSM13 (10^12^–10^13^ PFU/ml) (Stratagene) was added, and the cultures were shaken for an additional 1.5 h. Kanamycin (70 µg/ml) was added, and the cultures were shaken at 37°C overnight.

The cultures were spun down and phages were precipitated by addition of 4% (w/v) polyethylene glycol 8000 and 3% (w/v) NaCl, followed by incubation on ice for 30 min, and centrifugation at 37°C. Phage pellets were resuspended in 2 ml TBS with 1% BSA and microcentrifuged at room temperature for 5 min to pellet debris. The supernatant was sterilized by passing it through a 0.22-µm filter and stored at −20°C. This phage display antibody library was used for the following antigen panning.

### Selection of binding phage on immobilized rabies virus

The library was subjected to five rounds of panning, as previously described [14]. Before being selected with rabies virus, the phages were incubated with 1×10^6^ human cells for non-specific binding, and then panning with rabies virus protein. The phage library was incubated with 3% BSA for 30 min at room temperature and transferred onto microplates (Corning, NY, USA) coated with immobilized inactivated whole viruses of rabies virus CTN strain, at 0.5 µg/well, for 1 h at 37°C. Unbound phages were washed off with PBS/0.2% Tween-20 for 10–20 times. Antigen-bound phages were eluted using 0.5 ml trypsin/EDTA. The eluted phages were used to infect 2 ml fresh (OD_600_ = 0.8) *E. coli* XL1-Blue cells for 15 min at room temperature, and 10 ml prewarmed SB medium that contained 20 µg/ml ampicillin and 10 µg/ml tetracycline was added. The cultures were then shaken for 1 h at 37°C. Further growth, phage preparation, and panning were repeated as outlined above. After five rounds of immobilized antigen selection, random monoclonal phages were selected and screened by phage ELISA.

### Monoclonal phage ELISA

Specificity of individual phage Fab and soluble Fab were assessed by ELISA [15]. EIA/RIA Stripwell (Corning, NY, USA) 96-well plates were coated overnight at 4°C with fixed rabies virus protein of CTN strain (5 µg/ml), blocked with 1% BSA blocking buffer, and incubated. The eluted phages from the fifth round of panning were used to infect *E. coli* XL1-Blue cells and spread on LB plates with 50 µg/ml ampicillin and incubated at 37°C overnight. Single clones were selected randomly to produce phage as described previously. Fifty microliters of single phage preparation was added and incubated at room temperature for 1 h. As the negative control, empty phage was used. After washing twice with wash buffer (PBS with 0.05% Tween-20), for phage ELISA, 50 µl horseradish peroxidase (HRP)-conjugated anti-M13 monoclonal antibody (Amersham, Piscataway, NJ, USA) in milk blocking buffer (1∶2,000 dilution) was added for 1 h at room temperature. The reaction was visualized with TMB and H_2_O_2_ substrate, and stopped by 2 M H_2_SO_4_. Plates were read for OD_450_ with a reference wavelength of 630 nm, on a multiscan spectrum (Thermo Labsystems, USA).

### DNA sequence and analysis

High-titer clones were selected and cultured overnight, and the plasmids were extracted and sequenced. The DNA sequence of each Fab clone was analyzed with DNAclub and V-BASE2 software online.

### Fab expression and purification

The gene for pComb3XSS-Fab, which was confirmed as the correct sequence by DNA sequencing, was transformed into *E. coli* Top10F' (Invitrogen, Carlsbad, CA, USA) for expression [16]. Cultures of recombinant bacteria were induced with 1 mM IPTG (Biosharp) and cultured with shaking at 25°C for 12 h. The cultures were harvested by centrifugation at 4°C and the cell pellet was suspended in 200 ml PBS. After sonication, the supernatant was collected by centrifugation for 30 min (12,000 rpm) at 4°C, and analyzed for soluble expression of Fab.

The Fab fragment was purified from the supernatant and medium by affinity chromatography with an ImmunoPure Immobilized Protein L column (Pierce) using an FPLC system (Amersham Pharmacia Biotech, Uppsala, Sweden). Fab was eluted with glycine buffer (pH 2.8). The eluted fractions were concentrated by centrifugal filters (10,000 MWCO; Millipore, Bedford, MA, USA). The prepared Fab fragment which has neutralization activity (see below) was named as Fab094.

### Western blotting analysis

Protein samples were analyzed by electrophoresis on 10% SDS-PAGE under reducing conditions, and transferred onto nitrocellulose membranes. The membranes were first blocked by incubation with 5% nonfat milk and then with HRP-conjugated anti-human IgG (Fab-specific), and finally developed using the ECL detection system and exposed to X-ray film.

### Mass spectrometry (MS)

Rabies virus strain CTN proteins were mixed with 20 µg purified Fab094 and 50 µl protein-L Sepharose beads (Invitrogen), and incubated at 4°C overnight with gentle shaking. The immune complexes were detected by 12% SDS-PAGE under reducing conditions and transferred onto nitrocellulose membranes. The commercial mouse anti-rabies virus glycoprotein antibody C86307M, which reacts with a glycoprotein of rabies, more than 20 different strains from 4 serogroups, including CVS, Lagosbat, Mokola, Duwenhage were positive in neutralization (Meridian Life Science, USA), was added to the membranes for 1 h at room temperature. The HRP-conjugated goat anti-mouse IgG was added for another 1 h. Following further washes, bound antibodies were detected by the addition of the mixture of H_2_O_2_ and DAB, and after incubation for 15 min at 37°C, the reaction was stopped by washing with water.

The bands that corresponded to CTN virus protein on the polyacrylamide gel were analyzed by MS. Each gel slice was dissolved in 0.1% trifluoroacetic acid (TFA), desalted, and concentrated using ZipTips from Millipore. Peptide solution (0.5 µl) was mixed with 0.5 µl matrix (5 mg/ml α-cyano-4-hydroxycinnamic acid in 30% acetonitrile/0.1% TFA), spotted on a target disk, and allowed to air dry. Samples were analyzed by MS (Bruker Daltonics, Leipzig, Germany). Protein database searching was performed with the MASCOT search engine (http://www.matrixscience.com; Matrix Science, UK).

### Immunofluorescence assay (IFA)

Binding of Fab094 with rabies-virus-infected cells was determined by IFA. Sub-confluent BHK-21 cells, which were grown in six-well chambers, were infected with rabies virus strain CVS-11 at a multiplicity of infection of 0.1. After incubation for 24 h, the chambers were washed twice with PBS/Tween 20. Diluted Fab094 was added to virus-infected BHK-21 cells. After incubation at room temperature for 2 h, followed by three washes in 0.5% PBS/Tween 20, FITC-labeled anti-human IgG (Fab-specific) was added at dilution of 1∶100, and the cells were observed under fluorescence microscopy. The uninfected cells were used as a negative control. The experiments that involved the use of rabies strain CVS-11 were performed in a BSL-3 laboratory.

### Preparation of Fab094-CPNPs

Fab094-CPNPs were prepared using the adsorption technique [4]. Nanoparticles were prepared by a simple interfacial deposition method (nanoprecipitation). Briefly, 160 mg CaCl_2_, 160 mg NaH_2_PO_4_ and 160 mg sodium citrate were added to 180 ml distilled water under magnetic stirring at room temperature for 48 h, sonicated for 1 h, centrifuged (10,000 rpm, 15 min, 4°C), and the precipitate was separated. After centrifugation, the precipitate was resuspended in PBS, and sonicated for 1 h. Then, 10 mg Fab094 was added and stirred with the above suspensions to absorb the Fab094 at 4°C for 24 h. The resultant suspension was centrifuged and the precipitate was resuspended in pure water. The protein concentration of the supernatant was determined. Sizes of nanoparticles and Fab094-CPNPs were measured by Zetasizer-Nano instrument (Malvern, UK).

### Neutralization activity detection of Fab094 and Fab094-CPNPs *in vitro*

The sample was diluted three fold with DMEM that contained 10% FBS, and placed in a well. The samples were set up in duplicate. CVS-11 (100 TCID_50_) was added to each well and incubated in a 5% CO_2_ incubator at 37°C for 90 min. BSR cells were added to each well and incubated for 24 h. Finally, cells were fixed with 80% acetone and stained with FITC-conjugated anti-rabies N monoclonal antibody at 37°C for 30 min, and observed under a fluorescence microscope. Standard anti-rabies serum (30 IU/ml) was used as positive control. The average of duplicate samples was determined. The neutralizing antibody titer was calculated by the Reed–Muench method [17], [18].

### *In vivo* Kunming mouse challenge model

A lethal animal model that mimicked rabies exposure was used as described previously [19]–[22]. Kunming mice (17 groups of eight mice, 10–12 g) were infected with 100LD_50_/0.05 ml rabies virus CVS-11. Three hours later, prophylaxis was initiated with vaccine (diluted with PBS; Chiron Behring Vaccines) alone, single antibody (Fab094 or Fab094-CPNPs) alone, vaccine plus HRIG (20 IU/kg; Taibang Ltd., China), or vaccine plus 40, 32, 20, 8, 2 or 0.5 IU/kg single monoclonal antibody (Fab094 or Fab094-CPNPs). As a negative control, one group was treated with PBS. On day 7, mice were vaccinated with rabies vaccine again, except for the negative control group. The mice were examined daily for clinical signs of rabies and death. The mice were maintained and evaluated at up to 28 days after infection. The experiments that involved the use of rabies strain CVS-11 were performed in a BSL-3 laboratory. At necropsy, brain impressions were made and tested for rabies virus antigen by the direct fluorescent antibody test [16], [20].

### Data analysis

All data were processed and analyzed by SPSS10.0 Data Editor (SPSS Inc., Chicago, IL, USA). Fisher's exact test was used. The results in comparisons between groups were considered different if *P* was <0.05.

## Results

### Fab library construction and immobilized antigen panning

The Fab genes were successful amplified after three-step PCR (data not shown). The pooled Fab DNA was digested efficiently with *Sfi*I and cloned to pComb3XSS, and transformed into *E. coli* XL1-Blue to create a phage display antibody library with a capacity of 6.7×10^8^.

**Figure 1:**
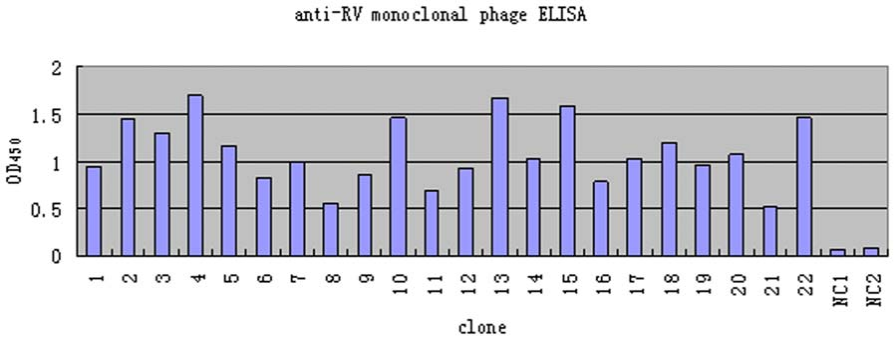
Fixed rabies virus protein specifically recognized colony- screening-positive phage clones in ELISA. Sixty individual bacterial clones were selected randomly after the fifth round of selection, to produce recombinant phages. As the negative control, empty phage was used. These phages were tested for their ability to bind to rabies virus protein by ELISA. Lane 1–22 were the representative clones of the 60, which had higher OD_450_ value. Lane NC 1–2 were empty phages.

After five rounds of panning, 60 individual phage clones were selected randomly and amplified to test for specific binding to rabies virus, by phage ELISA (Figure 1). As shown in Fig. 1, 22 clones were representative clones of the 60, which had higher OD_450_ value, and analyzed by DNA sequencing and BLAST analysis, which indicated that four unique phages (named Fab092, Fab093, Fab094, Fab095) encoded the different Fab DNA sequences. Unlucky, only Fab094 has the neutralization activity and Fab 094 DNA sequence has been deposited in GenBank (the accession numbers: VH was HQ706884 and VL was HQ706885).

### Fab expression and purification

**Figure 2:**
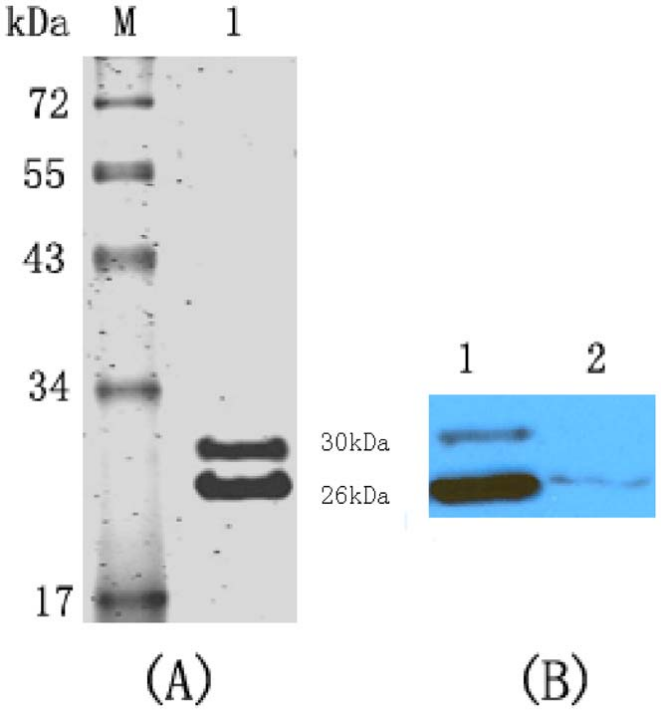
SDS-PAGE and western blotting analysis of the protein of purified Fab094. (A) Proteins were separated by 12% SDS-PAGE and stained with Coomassie Brilliant Blue. Lane 1 shows the protein of purified Fab094. Lane M contained the molecular-mass markers. (B) Western blotting analysis of Fab094 with HRP-conjugated anti-human IgG (Fab-specific). Lane 1 shows the ultrasonic supernatant of Fab094 with HRP-conjugated anti-human IgG; lane 2 was the culture supernatant of Fab094 with HRP-conjugated anti-human IgG.

The soluble Fab094 was purified from the periplasm of the bacteria using protein L affinity purification. One liter of the bacterial cultures typically yielded approximately 2 mg of the finial purified Fab094 product. The purified Fab094 was verified by SDS-PAGE and western blotting, which showed two bands at about 30 and 26 kD (Figure 2).

### Co-immunoprecipitation and MS

**Figure 3:**
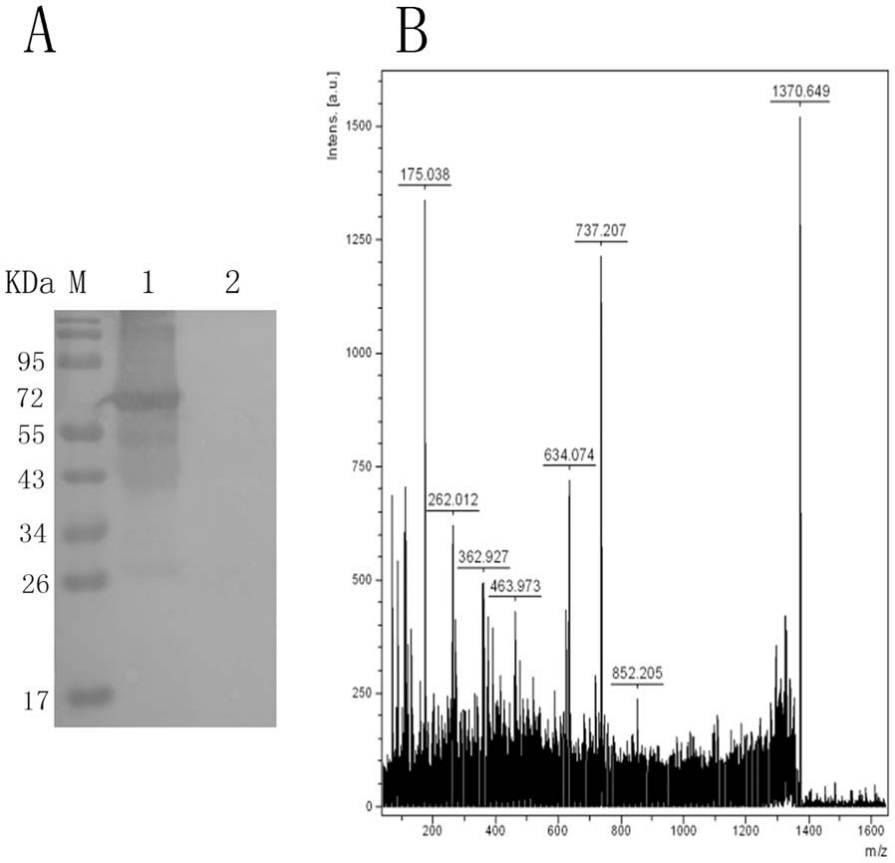
IP and MS analysis of Fab094 binding to rabies virus protein. (A) Proteins immunoprecipitated by Fab094 were separated by SDS-PAGE and probed with mAb(C86307M) by Western blot. (M): protein marker. (1): One protein was recognized by mAb(C86307M). The molecular weight of the protein was about 67 kDa. (2): BHK-21 lysate was used as the negative control to replace Fab094 in the IP. (B): MS spectrums of fragment ions were from the 67 kDa protein. Eight major (m/z = 175.038, 262.012, 362.927, 463.973, 634.074, 737.207, 852.205, 1370.649) ions were detected.

Immunoprecipitation was carried out with rabies virus strain CTN protein. Sixty-seven-kilodalton proteins were captured by Fab094, but they were not found in freeze–thaw lysates of BHK-21 cells, which are used routinely for rabies virus culture (Figure 3A). Eight peptide sequences (Table 1) matched with rabies glycoprotein by MS analysis (Figure 3B) were found when the identified peptides were compared with the known sequences of rabies glycoprotein in the SWISS PROT database.

**Table 1:** Amino-acid residue sequences of matched peptides.

	Relative intensity	Amino-acid residue
**1**	175.038	KHFRPTP DACR
**2**	262.012	YEES LHNPYPDYHW LR
**3**	362.927	LGTSCDI FTNSR
**4**	463.973	TCGFVD ER
**5**	634.074	LCGVLGLR
**6**	737.207	EECLDALESIM TTNPVSFRR
**7**	852.205	DGDE VEDFVEVHLP DVHK
**8**	1370.649	AESIQ HSFGETGRKV SVTSQSGRVI SSWESYK

### IFA

**Figure 4:**
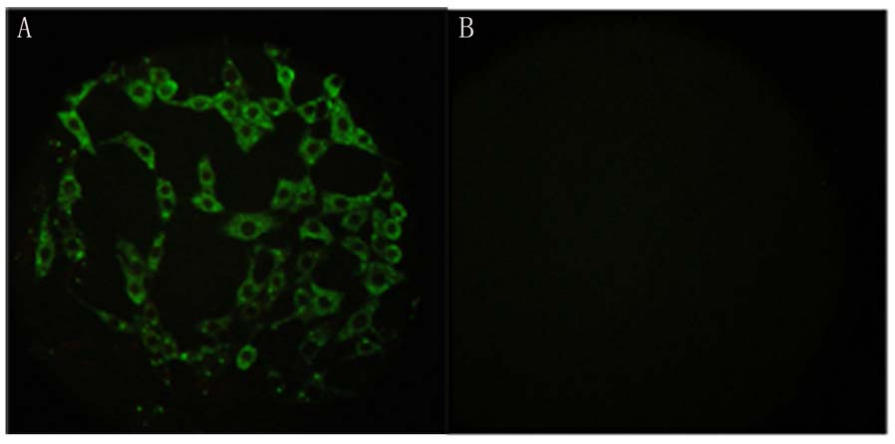
Detection of rabies-virus-infected BHK-21 cells by IFA. The slides were observed by fluorescence microscopy (400×). A: rabies-virus-infected cells stained with Fab094; B: normal BHK-21 cells with Fab094.

To ascertain whether Fab094 recognized rabies virus protein, virus-infected cells were incubated with Fab094 followed by FITC-labeled anti-human IgG (Fab-specific). The fluorescent antigens were detected on the surface of virus-infected cells (Figure 4). These findings suggested that Fab094 recognized the authentic rabies virus protein.

### Construction of Fab094-CPNPs

Four Fab antibody preparations were examined for neutralization activity against rabies virus strain CVS-11, but only Fab094 exhibited neutralizing activity (described below). Following, Fab094 was loaded calcium phosphate nanoparticles and named as Fab094-CPNPs. The average size of nanoparticle was 260 nm.

### Neutralization activity of Fab094 and Fab094-CPNPs *in vitro*

The Fab094 neutralizing activity measured by RFFIT was 200.11 IU/mg, while that of Fab094-CPNPs was 246.12 IU/mg. Fab092, Fab093 and Fab095 failed to show any neutralizing activity against the CVS-11 strain.

### Virus neutralizing activity *in vivo*

**Figure 5:**
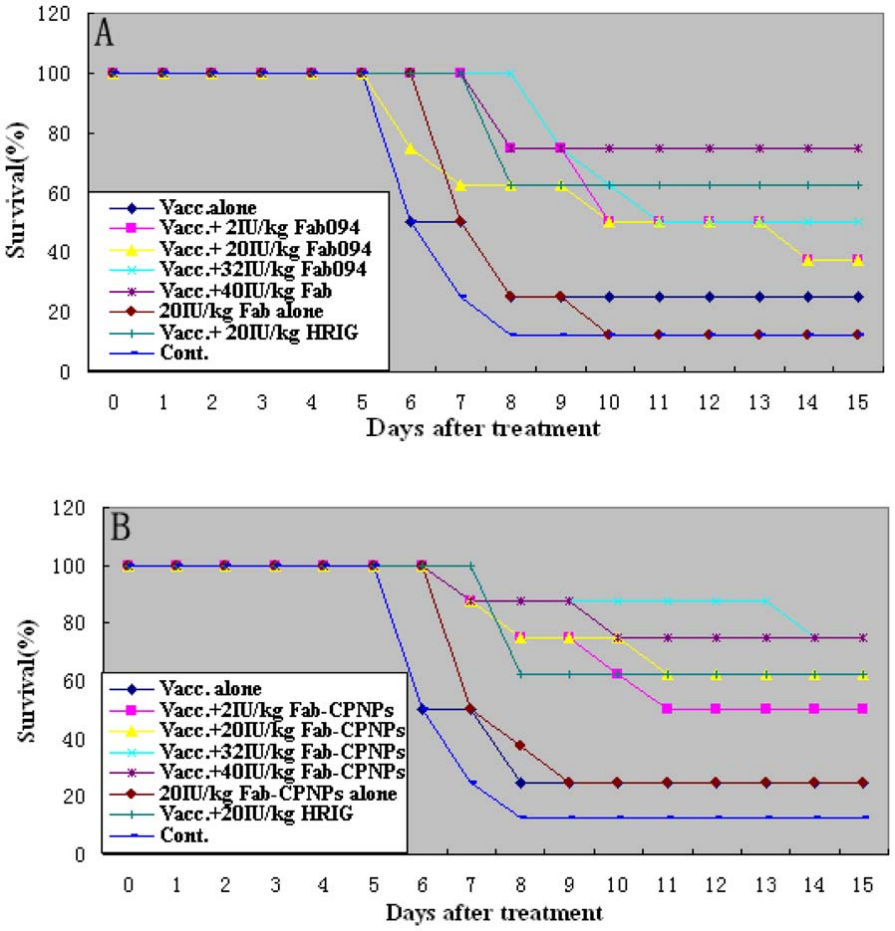
Kaplan-Meier survival curves for Kunming mouse after rabies virus challenge. Kunming mice (17 groups of eight mice, 10–12 g) were infected with 100LD_50_/0.05 ml rabies virus CVS-11. Three hours later, prophylaxis was initiated with vaccine (diluted with PBS; Chiron Behring Vaccines) alone, single antibody (Fab094 or Fab094-CPNPs) alone, vaccine plus HRIG (20 IU/kg; Taibang Ltd., China), or vaccine plus 40, 32, 20, 2 or 0.5 IU/kg single monoclonal antibody (Fab094 or Fab094-CPNPs). As a negative control, one group was treated with PBS. On day 7, mice were vaccinated with rabies vaccine again, except for the negative control group. The mice were examined daily for clinical signs of rabies and death. The mice were maintained and evaluated at up to 28 days after infection. The mice were monitored twice daily and were killed when clinical signs of rabies appeared. Kaplan-Meier survival curves are shown for days 0–15. The mice were monitored until day 28 after treatment (no additional deaths occurred between days 16 and 28).

The survival rate against CVS-11 infection is shown in Figure 5. Data indicated that 40 IU/kg Fab094 or 8 IU/kg Fab094-CPNPs (62.5%, figure data not shown) provided a level of protection against rabies comparable with that provided by 20 IU/kg HRIG. These data illustrated that Fab094 and Fab094-CPNPs had strong neutralizing potential, and Fab094-CPNPs had stronger neutralizing potential than Fab094 at an equivalent concentration. The survival rate in mice treated with Fab094 or Fab094-CPNPs alone was 12.5% and 25%, respectively. This result also showed that antibody without vaccine did not prevent rabies infection. Statistical analysis showed that the survival rates of groups with 40 IU/kg Fab094, 32 IU/kg and 40 IU/kg Fab094-CPNPs were significantly higher than control group (*P*<0.05). Necropsy of the brain showed that all mice had infection with rabies virus (data not shown).

## Discussion

Rabies virus entry occurs through wounds or direct contact with mucosal surfaces. For PEP, early local injection-site reactions that consist of erythema and itching are not uncommon with purified HRIG and ERIG. Published data indicate that immunoglobulins can be injected safely into already infected animal bite wounds after proper wound cleaning and administration of appropriate antibiotics [23]. Human monoclonal antibodies produced in accordance with industrial standards could provide a good solution to the current global shortage of HRIG [20]. To date, some antibody engineering technologies have been developed to achieve this goal: fully humanized antibodies are derived by immunizing transgenic mice, and selecting the recombinant human antibody native or immunized phage display libraries. In comparison to other technologies, antibody phage display has the advantages of being inexpensive and highly efficient. The study of antigen panning, sequencing and purification to obtain a specific antibody fragment typically can be completed within several weeks. We have successfully constructed a human immunized Fab phage library with a diversity of 6.7×10^8^, and that library was used in the present study to generate a neutralization Fab fragment against rabies virus.

In this study, we constructed an immunized phage display antibody library using RNA from human peripheral blood lymphocytes from 45 rabies-vaccinated volunteers. The neutralizing human Fab antibody fragments were selected from this library with whole rabies virus particles. Among the selected Fab clones, Fab094 revealed neutralizing activity against strain CVS-11 when tested by RFFIT. Immunoprecipitation and MS assay showed that the Fab094 fragments bound to the glycoprotein of CTN strain, which is epidemic in China [13].

The aim of the present study was to generate a human Fab fragment that recognized rabies virus glycoprotein and was able to inhibit virions binding to and entering humanized cells, therefore, the antigen panning strategy was crucial. To obtain phage clones of high specificity and affinity for rabies virus, we maximized the library density of phage to about 10^14^ pfu/ml, which is the highest concentration to which phage can be condensed for the first round of panning. This procedure yielded an average of 2.5×10^5^ clones. In addition, the wash step in the first round was not sufficiently stringent to elute phage for amplification, to increase the chance that all of the rabies-virus-binding phages were collected. If there were <10^5^ eluted phages, the specific phage might have failed to be enriched. The conventional panning method is to use recombinant antigen coated on a solid substrate; usually a microtiter plate. However, the antigen on the virus surface might be different from the purified protein because of the conformational changes, and this could decrease the chances of isolating phages that bind to the protein. In the present study, the whole virus particles used as a vaccine were used to coat the immunoplates for phage panning, to select the antibody that bound to the antigen in native comformation.

Several studies have confirmed that the glycoprotein is the important antigen of rabies virus; it is capable of inducing and binding neutralizing antibodies to the virus, which confer immunity against a lethal challenge infection with the virus [24], [25]. In the present study, western blotting (Fig. 2) and MS (Fig. 3) showed that Fab094 could bind with the 67 kD glycoprotein of rabies virus, which suggests that Fab094 might have the ability to neutralize rabies virus.

Ando et al. have described two rabies-virus-neutralizing Fabs isolated from a combinatorial human Fab phage display library, but two antibodies exhibited neutralizing activity with an infected cell count reduction of 76% or 57% at a dilution of 1∶2, and of 20% or 41% at a dilution of 1∶4 [1]. In the present study, RFFIT was used to measure Fab094 and Fab094-CPNPs. The data showed that the titration by RFFIT was 200.11 IU/mg (Fab094) and 246.12 IU/mg (Fab094-CPNPs). The data also indicated that Fab094-CPNPs had higher neutralization activity than Fab094 at an equivalent concentration.

Furthermore, *in vivo* studies indicated that treatment of Kunming mice with each rabies antibody resulted in protection equivalent to that offered by HRIG when mice were challenged with a lethal rabies virus dose. In the vaccine only group, the survival rate was low (25%). This could have been because the injection site of rabies was in the foreleg of the mice, which was close to the central nervous system and brain, or perhaps the virus had invaded the neurocytes before vaccine-induced antibody production. This result also suggests that vaccine alone cannot provide sufficient survival, and antibody must be used in category III PEP. Similarly, antibody alone did not provide sufficient protection. This might be related to antibody degradation *in vivo*. In mice treated with Fab094 and vaccine, the survival rates increased with dosage. A clear dose effect was observed in the mice treated with 40, 32, 20, 8, 2 and 0.5 IU/kg Fab094, which produced survival rates of 75%, 50%, 37.5%, 37.5%, 37.5% and 37.5%, respectively. This indicated that 40 IU/kg Fab094 provided a level of protection against rabies comparable with that provided by 20 IU/kg HRIG, which illustrated the strong neutralizing potency of Fab094. For Fab094-CPNPs, 8 IU/kg antibody plus vaccine provided a protective rate that was equal to that with 20 IU/kg HRIG plus vaccine. The reasons for this phenomenon should be studied further. Taken together, these results indicate that the human Fab094 and Fab094-CPNPs, especially the latter, might be efficacious candidate drugs to replace RIG for rabies PEP.

In conclusion, the current study describes the isolation of human Fabs with rabies-virus-neutralizing activity from a human immunized phage display library using peripheral blood lymphocytes from 45 rabies hyper-immune volunteers in China. In addition, we developed Fab094-CPNPs and tested their efficacy by *in vitro* neutralization assay. This conclusion was confirmed by an *in vivo* Kunming mice challenge model. These results demonstrate that human Fab094 and Fab094-CPNPs might be efficacious candidate drugs to replace RIG for rabies PEP.
